# The Squamous Palmar Syphilide*Read at Waco Meeting, Central Texas Medical Association, July 11, 1899.

**Published:** 1899-09

**Authors:** A. H. Ohmann-Dumesnil

**Affiliations:** St. Louis


### The Squamous Palmar Syphilide.*

BY DR. A. H. OHMANN-DUMESNIL, ST. LOUIS.

The squamous palmar syphilide is among the most interesting of cutaneous affections. "Syphilitic psoriasis" is a misnomer, as syphilis and psoriasis have no relation to each other. They may co-exist, however.

This trouble depends entirely upon the luetic process without reference to or dependence upon any other disease. In fact it is a pathognomonic lesion of syphilis. Analagous lesions may appear on the soles of the feet, which are the analogues of the palms. The anatomical structure of the integument of the palms and soles is identical in man, as they positively are in the quadrumana. This is the reason that a generalized disease like lues w'll produce the same effect upon similar structures at the same time. The peculiarity of the structure of the skin has much to do with the form of involvement which manifests itself. This eruption is a late secondary manifestation. Its appearance is uncertain as to time after the appearance of chancre.

It is not, by any means, a necessary one in every case of lues, and may not appear in neglected cases, but these are the ones in which it generally does appear. It may appear on palms alone, or also on the fingers, and also on forearm and face. Lesions are larger on the face, and the squamous conditions are more marked. The process is symmetrical in its manifestations. In another variety the squamous lesions are small, but plainly marked. They are fairly numerous on the palms, and continue on the forearms, as well as on the fingers. On the face some may be seen mixed with flat papular syphilides about to become squamous. They have a tendency to group themselves in the form of the segments of circles, more especially on the face, and resemble psoriasis. These cases have not generally been treated, and the disease first evidences the secondary phase in the form of a roseola which was quickly followed by a papular syphilide, to which succeeded the squamous form.

The next form, confined to palms and soles, has been called "syphilitic corns," from its fancied resemblance to a corn; but it is not dependent on friction or pressure on the palm. Keratomata may exist and may be dug out with the point of a pen knife, but they

*Read at Waco Meeting, Central Texas Medical Association, July 11, 1899.

immediately recur unless means for total eradication be adopted. Lesions are not confined to workingmen; they are confined entirely to the palms and soles.

DIFFERENTIAL DIAGNOSIS.—Psoriasis of palms is rare. Some authorities deny that it exists: others *per contra* have maintained that they have observed it. We are disposed to accept the latter statement, notwithstanding we never observed an undoubted case. The difference is in the following points: In syphilis there is history of luetic infection, and concomitant corroborative symptoms may be found. In psoriasis there exist lesions on other parts of the body, and the palms alone will be implicated, the soles not. Knees and elbows will likely participate in the process. The buttocks, and back, too, are liable to have the characteristic lesions of psora. But if lesions are confined to palms, the history of the case should furnish more definite information. The itching present precludes the thought of syphilis. In psoriasis there is a constant increase of the epithelial horny cells, which assume a silvery, shining appearance, not seen in syphilis, and the inflammatory condition is evidenced by the areola surrounding each lesion.

There is no occasion for an error in diagnosis. Squamous eczema (of palms) may be mistaken for squamous palmar syphilide and *vice versa.* Eczema, like the syphilide, attacks the soles of the feet, and faintly resembles the localization of the specific trouble. In eczema the keratization is more marked; the horny layer is much thicker. Larger plaques appear, and have a tendency to coalesce and involve the entire palm or sole, conditions not present in syphilis. Generally, a certain amount of itching attends eczema, and there is always a tendency to the formation of fissures, and these grow deeper and deeper, exposing the corium, and having thick, everted edges. The fissures are painful, and any foreign body, as well as the air, being a constant source of irritation. Mechanical pressure of muscular contractions and extensions lead to the widening of these fissures. Fissures occur along the natural folds of the palm. History of the case will aid in making correct diagnosis. Syphilitic horns, from a pathogenic point of view, are allied to squamous syphilide. It is rare, and is merely mentioned here because it is a keratogenous manifestation.

The length of duration of squamous palmar syphilide is indefinite, unless proper therapeutic measures be employed. Internal treatment seems impotent to cause disappearance of local lesions, unless given during the existence of the flat papules, and before the squamous condition has asserted itself, then it may suffice; but when the scaly condition appears, local measures are necessary. Internal remedies will aid local treatment. A judicious combination of local and general treatment will prove most satisfactory. Some cases demand active mercurization. To begin with, mercurial treatment alone, or mixed treatment, is indicated. A good plan is to give about twenty mercurial injections followed by mercauro in 15-drop doses thrice daily, and slowly increase until 25 or 30 drops are given at a dose. If frictions are objected to, then begin with bin-iodide of mercury in ¼-grain doses, thrice daily. Thus give about sixty pills. Then mercauro is to be given as above stated. A valuable mixed treatment in cases somewhat advanced in their evolution is:

℞ Hyd. ch. coros.......................gr. jv

Kali iodide..............................℥j

Aq. dest.................................℥iij

Syr. Sarsaparilla eo.....................℥j

M. Sig.: A teaspoonful in water after each meal.

This is very efficient and not particularly irritating. In some females any of the above methods may prove irritating, when recourse must be had to the proto-iodide of mercury, and if this is also not to be considered, hypodermic injections of mercurials are to be used. Whatever preparation is employed must be continued long enough to obtain beneficial results.

Local treatment is about the same as would be indicated in a similar kerato-dermic affection of a non-specific origin. We may use plasters, ointments or films. *Emplastum acid salicylic* may be put on the palms, but it produces pain in a very short time. For this reason, also, the preliminary scrubbing with green soap is not to be recommended. The following ointment is both acceptable and efficient:

℞ Acid Salicylic.........................℈j

Ichthiolis...............................ʒss

Ung. aquæ rosæ...........................℥j

M. Sig.: Apply thoroughly twice a day.

If ointments are objected to, the following will prove efficient. It is dry, water-proof and not easily removed by friction:

℞ Acid Salicylic.........................℈j

Cocaini muriatis.........................gr. jv

Traumaticini.............................℥j

M. Sig.: Paint on affected parts twice daily.

If objection is made to the dark color of the preparation the flexible collodion can be substituted as the vehicle. Another set of the local applications are the varnishes, which have the disadvantage of requiring more time to dry. A very good form to use is that in which the tincture of benzoin is the vehicle.

Any local treatment must be faithfully applied if good results are expected. After the lesions have disappeared a bland ointment should be applied at night. The following is good:

℞ Hydy. ch. mite............................ʒss

Ung. aquæ rosæ.............................℥j

M. Sig.: Apply thoroughly at night. A rapid return to the normal should follow the above course of treatment.

It will surprise many to find that such simple measures will prove successful where active internal treatment has failed.

